# Contrafreeloading in kea (*Nestor notabilis*) in comparison to Grey parrots (*Psittacus erithacus*)

**DOI:** 10.1038/s41598-022-21370-6

**Published:** 2022-10-18

**Authors:** Gabriella E. Smith, Amalia P. M. Bastos, Martin Chodorow, Alex H. Taylor, Irene M. Pepperberg

**Affiliations:** 1 The Alex Foundation, 30 Curry Circle, Swampscott, USA; 2 Messerli Research Institute, University of Veterinary Medicine Vienna, Vienna, Austria; 3 School of Psychology, The University of Auckland, Auckland, New Zealand; 4 Department of Cognitive Science, University of California San Diego, San Diego, USA; 5 Department of Psychology, Hunter College, The City University of New York, New York, USA; 6 Institut de Neurociències, Universitat Autònoma de Barcelona, Barcelona, Spain; 7 ICREA, Pg. Lluís Companys 23, Barcelona, Spain; 8 Department of Psychological and Brain Sciences, Boston University, Boston, USA

**Keywords:** Behavioural ecology, Animal behaviour

## Abstract

Contrafreeloading—working to access food that could be freely obtained—is rarely exhibited and poorly understood. Based on data from Grey parrots (*Psittacus erithacus*), researchers proposed a correlation between contrafreeloading and play: that contrafreeloading is more likely when subjects view the task as play. We tested that hypothesis by subjecting a relatively more playful parrot species, the kea (*Nestor notabilis*), to the same experimental tasks. Experiment 1 presented eight kea with container pairs holding more- or less-preferred free or enclosed food items, and examined three types of contrafreeloading: calculated (working to access preferred food over less-preferred, freely available food); classic (working to access food identical to freely available food); and super (working to access less-preferred food over preferred, freely available food). At the group level, the kea behaved similarly to the Greys: They significantly preferred calculated contrafreeloading, performed classic contrafreeloading at chance, and significantly failed to super contrafreeload. However, overall kea engaged in more contrafreeloading than Greys. Experiment 2 examined a potentially more ecologically relevant task, a choice between shelled and unshelled walnuts. No kea contrafreeloaded for nuts, whereas two of five Greys significantly preferred nut contrafreeloading and one chose at chance. We examine proximate and adaptive explanations for the performances of these differentially playful parrot species to further elucidate the role of play in contrafreeloading.

## Introduction

According to optimal foraging and standard learning theories (e.g.,^1–3^), contrafreeloading—working to access food that could be consumed for free^4^—should not exist. In fact, some researchers contend that the behaviour is so unlikely that *any* evidence of its existence is of interest (e.g.,^5^). Several theories have been proposed to provide some rationale for reported instances of contrafreeloading, such as conditioned reinforcement (e.g.,^6^), work ethic (e.g.,^7^), relief from boredom (e.g.,^8^), predispositions for ethologically relevant behaviour^9^, and information primacy theory (e.g.,^10^), but no single theory appears to fully explain the phenomenon (for reviews of most of these theories, see^11,12^). Smith and colleagues explored an additional explanation in a study of contrafreeloading in Grey parrots (*Psittacus erithacus*) living in an enriched environment with ad libitum food—the role of play^13^. Their definition of play was based on previous studies in which play was “characterized by positive mood, intrinsic motivation, occurring in a protected context and easily disrupted by stress”^14^, and as varying between individuals of the same species, often composed of incomplete or modified, repeated, and seemingly non-functional actions^14,15^. Using this definition, Smith et al. argued that contrafreeloading is correlated with play; that is, contrafreeloading is likely to occur if an individual characterizes the activity involved as play-like, rather than work-like; they further proposed that each individual’s categorization affects the extent of its contrafreeloading^13^.

Smith et al. tested this hypothesis with Grey parrots in two different contrafreeloading experiments^13^. Experiment 1 presented subjects with pairs of more-, less-, or equally-valued food rewards placed in lidded or unlidded cups (i.e., the latter providing freely available food); types of contrafreeloading were defined as calculated (working to access preferred food over less-preferred, free food), classic (working to access food identical to the free food), or super (working to access less-preferred food over preferred, free food). Birds were also tested on their proclivity to engage in the task in the absence of any food reward. Experiment 2 involved contrafreeloading for nuts as the reward, hereafter nut contrafreeloading (choosing to crack a nutshell to access the nutmeat inside over an identical, already-shelled nut; for Greys, a more ecologically relevant task). These different forms of contrafreeloading are summarized in Table 1.

**Table 1:** Contrafreeloading type definitions with examples.

Type	Definition	Example(choice of lidded/shell option)
Calculated	Performing an activity to access preferred food over less-preferred, free food	Fat (lidded) versus sultana (unlidded) Fat (lidded) versus hazelnut (unlidded) Hazelnut (lidded) versus sultana (unlidded)
Classic	Performing an activity to access food identical to the free food	Fat (lidded) versus fat (unlidded) Hazelnut (lidded) versus hazelnut (unlidded) Sultana (lidded) versus sultana (unlidded)
Super	Performing an activity to access less-preferred food over preferred, free food	Hazelnut (lidded) versus fat (unlidded) Sultana (lidded) versus hazelnut (unlidded) Sultana (lidded) versus fat (unlidded)
Nut	Performing an activity to access nut in shell over nut without shell	Nut (with shell) versus nut (without shell)

Decisions to participate in any type of contrafreeloading varied considerably across individuals. Smith et al. hypothesized that the extent of contrafreeloading performed by each individual may have reflected how they categorized the value of the actions involved in the task itself, or relative to that of the food available (see within-trial contrast theory^16^), and thus that Grey parrots contrafreeloaded when the task was viewed as a form of play rather than work^13^. To reiterate: They did not claim that contrafreeloading could be explained solely on the basis of the action involved being viewed as play. They did, however, hypothesize that, given that contrafreeloading was not *fully* explained in their study by any one of the other previously proposed theories noted above, the concept of an individualistic evaluation of play should be considered as a heretofore overlooked contributing factor^13^.

Smith et al.’s hypothesis thus was based on the extent to which proximate values for play and contrafreeloading potentially overlap^13^—for example, those of being intrinsically rewarding^4^ and adding to a reward’s worth^17^, assisting in information gathering^10^, and how contextual variables, such as the effect of physical and mental stress, affect both behaviours^14,18,19^. The question remains, however, as to why contrafreeloading and play exist at all; that is, what are their ultimate values? Is an overlap between play and contrafreeloading indicative of some shared evolutionary adaptation? Historically, the adaptive explanation for play maintains that its expression should be ultimately beneficial to survival (e.g., play as practice for adult foraging or fighting^20–23^), but this view fails to account for behaviours that lack apparent adaptive or functional purposes [e.g., stone play in macaques (*Macaca fuscata*)^24^; stick-weaving in tamarins (*Saguinus oedipus*)^25^; thermometer jousting in cichlid fish (*Tropheus duboisi*)^26^], or behaviours that could be executed with less energetic expenditure^15^. Possibly, the adaptive purpose of playing—and hence also of contrafreeloading—may instead be to enhance executive function and general cognitive processing^27^.

Smith et al.’s hypothesis that contrafreeloading is in fact related to, and therefore can be predicted to some extent by, play could be tested by comparing species that are expected to exhibit different amounts of play. If contrafreeloading occurs as a natural extension of or is correlated with play, then species that engage in more frequent and/or complex play should also contrafreeload to a greater extent than species that play less or more simply. Research already exists that compares the playfulness of different avian species, such as that of Auersperg and colleagues, which compared frequency and types of object play in Psittaciformes and corvids and found considerable evidence that play behaviour was better explained by the species’ ecological specializations than by phylogeny^28^. Specifically, ravens (*Corvus corax*), known for food caching, frequently cached play objects, whereas New Caledonian crows (*Corvus moneduloides*) and Goffin’s cockatoos (*Cacatua goffini*) most often combined objects in ways reminiscent of their physical problem-solving skills. In contrast, Grey parrots engaged in comparatively little object play^29^; however, their physical cognition (e.g., tool use), remains mostly untested and their contrafreeloading, although non-negligible, was not widespread and varied considerably across individuals and tasks^13^. The question remains as to the level of contrafreeloading exhibited by other, more playful, parrot species.

The kea (*Nestor notabilis*), a species that engages in both social and object play throughout its life^30–32^ and even produces a positive emotionally contagious call that occurs during playful interactions^29^, offers an ideal first point of comparison to investigate a possible relationship between play and contrafreeloading behaviour. Thus, we here test kea on Smith et al.’s two experiments^13^, examining the exact same forms of contrafreeloading (calculated, classical, super, nut). Noting the individual differences observed in Grey parrots^13^, we also test for individual as well as group differences in contrafreeloading in the kea.

We reiterate that contrafreeloading is a surprising and quite uncommon behaviour^5,13^. For that reason, we did not expect to find such activity at *statistically significant* levels in kea, particularly given that the tasks, specifically chosen for the Greys, may or may not be appealing to the kea. Nor—more importantly—did we actually expect to find *statistically significant* differences between kea and Greys, again because the tasks may have had less appeal for the kea, but also because trying to claim statistically valid correlations between what would likely be non-statistically significant values for their actions would make little sense. Based on our hypothesis that contrafreeloading and play are correlated, we simply predicted that for the given tasks, the kea, when compared to the Greys, should perform contrafreeloading qualitatively more often and that a higher percentage of individuals would engage in the behaviour.

## Results

### Experiment 1

#### Empty cup controls

In 20 lid preference trials performed at the start of the experiment, subjects chose between empty lidded and unlidded cups. This control sought to test whether kea exhibited any natural preferences for or against lid-popping, prior to experiencing any experimental trials. Given their experimental background and extent of daily environmental enrichment^33,34^, we needed to determine if they would engage in the task at all. Only two subjects showed a statistically significantly preference for the lidded cup (Neo and Plankton, see Table 2), but all eight subjects chose the lidded cup at least half the time (sign test, *p* = .016), indicating, at the group level, a preference for popping lids off the empty cups. A logistic regression revealed no significant change in lid-popping preference as a factor of trial number at the group (*p* = .80) or individual (all *p*’s > .13) level.

**Table 2:** Individual preferences for the lidded cup when both cups were presented empty.

	Choice of lidded empty cup
Blofeld	13/20
Bruce	11/20
Harley Quinn	10/20
Loki	14/20
Moriarty	11/20
Neo	18/20***
Plankton	17/20**
Taz	13/20

**p < 0.01**,** ***p < 0.001.

#### Food preferences

In food comparison trials (fat, hazelnut, sultana) where both cups were either lidded or unlidded, but paired foods varied in value, subjects continued to select their preferred food in 89.1% of trials. As the experiment progressed, the probability of fat being selected over other food types increased significantly (B = 1.950, SE = 0.448, *p* =  < 1.36e^−05^, 95% CI [1.140, 2.917]), but no significant change occurred for hazelnut (*p* = 0.296) or sultana (*p* = 0.392). These data indicate that the kea had consistent relative food preferences over the course of the experiment.

#### Contrafreeloading

At the group level, results varied by type of contrafreeloading. Subjects displayed more calculated contrafreeloading than expected by chance, where effort was expended for the higher food value (B = 2.152, SE = 0.193, *p* < 2e^−16^, 95% CI [1.791, 2.550]), and they contrafreeloaded significantly less often in classic and super contrafreeloading trials compared to calculated contrafreeloading (see Table 3). Releveled models in which each of the other trial types serves as the reference level revealed that, at the group level, classic contrafreeloading occurred significantly less often than chance (B = − 0.251, SE = 0.119, *p* = 0.034, 95% CI [− 0.486, − 0.019]), as did super contrafreeloading (B = − 2.44, SE = 0.217, *p* < 2e^−16^, 95% CI [− 2.898, − 2.042]).

**Table 3:** Logistic regression model for contrafreeloading choices for the three trial types (calculated, classic, and super contrafreeloading).

Contrafreeloading type	B(SE)	Pr( >|z|)	95% CI for odds ratios		
			Lower	Odds ratio	Upper
Intercept(calculated)	2.152 (0.193)	< 2e^−16^***	5.997	8.600	12.809
Classic	− 2.403 (0.227)	< 2e^−16^***	0.057	0.090	0.139
Super	− 4.596 (0.291)	< 2e^−16^***	0.006	0.010	0.017

***p < 0.001.

At the individual level, all kea performed calculated contrafreeloading significantly above chance, classic contrafreeloading at chance, and super contrafreeloading significantly below chance (Table 4). This trend was comparable to the results from the original Grey parrot study^13^, where three of four subjects exhibited only calculated contrafreeloading significantly above chance (one exhibited both classic and calculated contrafreeloading significantly above chance), and none performed super contrafreeloading to a significant extent. As in Smith et al.^13^ it is important to note that even though kea did not perform classic contrafreeloading to a statistically significant extent, the behaviour was still performed by all subjects^13^. Blofeld and Bruce, for example, engaged in classic contrafreeloading half the time, and all other birds more than a third of the time. In contrast, very few engaged in super contrafreeloading; Bruce and Blofeld, who did so most often, each performed the behaviour only 6/36 times (17%).

**Table 4:** Subjects’ performances in calculated, classic, and super contrafreeloading trials.

	Calculated	Classic	Super
Blofeld	34/36***	18/36	6/36***
Bruce	30/36***	18/36	6/36***
Harley Quinn	30/36***	14/36	1/36***
Loki	35/36***	14/36	2/36***
Moriarty	30/36***	14/36	4/36***
Neo	34/36***	15/36	0/36***
Plankton	32/36***	16/36	2/36***
Taz	33/36***	17/36	2/36***

***p < 0.001.

#### Contrafreeloading without consumption

Of the eight kea, Bruce was the least likely to consume the food after popping a lid, failing to eat in 13/18 trials (72%) in which he classically contrafreeloaded, and was more likely not to eat in trials involving sultana (8/9) than hazelnut (4/4) or fat (1/5). When he performed calculated contrafreeloading, he did not consume the food underneath the lid 8/30 times (27%) [6/10 hazelnut (closed) vs sultana (open); 1/10 fat (closed) vs sultana (open); 1/10 fat (closed) vs hazelnut (open)]. After super contrafreeloading, he did not consume the food in the cup 4/6 times (67%) [3/3 sultana (closed) vs hazelnut (open); 1/1 sultana (closed) vs fat (open); 0/2 hazelnut (closed) vs fat (open)]. As for the Greys, contrafreeloading without consumption was relatively rare, but when it occurred, the discarded food item was also most often the least preferred.

#### Effect of trial number

Trial number did not significantly improve the fit of the model shown in Table 2, suggesting that at the group level, contrafreeloading behaviour did not significantly change over the course of the study. However, post-hoc individual-level logistic regressions with contrafreeloading type and trial number as predictors did reveal different patterns in contrafreeloading for three individuals as a factor of trial type and trial number. Runs tests and autocorrelation tests at lags 1, 2, and 3 were used to check for sequential independence of the outcome variable, contrafreeloading. With increasing trials, Bruce showed a significant increase in calculated contrafreeloading (B = 0.026, SE = 0.011, z = 2.40, *p* = 0.016, 95% CI [0.009, 0.054]), Harley Quinn showed a significant reduction in classic contrafreeloading (B =  − 0.008, SE = 0.004, z =  − 2.022, *p* = 0.043, 95% CI [− 0.017, − 0.001]), and Blofeld showed a significant reduction in super contrafreeloading (B =  − 0.014, SE = 0.007, z =  − 2.117, *p* = 0.034, CI [− 0.032, − 0.003]). However, none of these effects of trial number was significant after false discovery rate adjustments for multiple tests. Similarly, none of the Greys in the original study displayed significant changes in contrafreeloading behaviour as a function of trial number^13^.

#### Food items paired with empty cups and various lid conditions

Finally, in trials in which lid-status was variable (one lidded and one unlidded), where only one cup contained food and one was left empty, the best-fitting model for these trials included only food location (in the lidded or unlidded cup), whereas food value (low, medium, and high-value rewards) did not significantly improve the fit of the model. Thus, kea were significantly more likely than chance to select the lidded cup with food and ignore the unlidded empty one (B = 1.825, SE = 0.170, *p* =  < 2e^−16^, 95% CI [1.503, 2.173]; Table 5), regardless of food type. Only Bruce and Harley Quinn did not consume the chosen food after popping the lid [respectively, 8/17 (47%) and 3/11 (27%) of these trials], doing so most often for their least preferred food (sultana). Similarly, the Greys predominantly chose freely available food in the unlidded cup over the empty lidded cup, and food in the lidded cup over an empty unlidded cup; if non-consumption was observed, the least-favourite food was discarded the most often. However, Greys occasionally chose the empty, lidded cup over free, least-preferred food. Two kea (Plankton and Taz) did so half the time and Bruce did so on two-thirds of his trials.

**Table 5:** Logistic regression model output for subjects’ preference of the lidded cup when it contained food, compared to when it was empty and the food was presented in the unlidded cup.

Choice of lidded cup when:	B(SE)	Pr( >|z|)	95% CI for odds ratios		
			Lower	Odds ratio	Upper
Food in lidded cup	1.825 (0.170)	< 2e^−16^***	4.496	6.200	8.784
Food in unlidded cup	− 3.538 (0.236)	< 2e^−16^***	0.018	0.029	0.046

***p < 0.001.

### Experiment 2

#### Nut contrafreeloading

In 20 trials, subjects chose between walnut halves with or without shells, offered in identical, unlidded cups. Like two of the Grey parrots^13^, all kea preferred the walnut without a shell significantly above chance, avoiding contrafreeloading (Table 6). Across individuals, contrafreeloading occurred 13 times in total, with Harley Quinn the most likely to do so (5/13). She was also the most likely to discard a choice (~ 44% of the time), but never when she contrafreeloaded.

**Table 6:** Individual subject preferences for walnut with shells over those without. Non-naturalistic classic contrafreeloading data (Experiment 1) are displayed for comparison.

	Choice of walnut with shell	Classic contrafreeloading in experiment 1
Blofeld	0/20***	18/36
Bruce	0/20***	18/36
Harley Quinn	5/20*	14/36
Loki	1/20***	14/36
Moriarty	0/20***	14/36
Neo	2/20***	15/36
Plankton	3/20**	16/36
Taz	2/20***	17/36

*p < 0.05, **p < 0.01, ***p < 0.001.

#### Comparisons between Experiment 1 and 2

Two-tailed Fisher’s exact tests comparing the naturalistic (nut) to non-naturalistic contrafreeloading (classic contrafreeloading in cups) revealed that seven of eight kea expressed a statistically significant preference for non-naturalistic classic over naturalistic nut contrafreeloading (*p*-values adjusted using false discovery rate test): Blofeld (*p* < 0.001); Bruce (*p* < 0.001); Loki (*p* = 0.016); Moriarty (*p* = 0.002); Neo (*p* = 0.022); Plankton (*p* = 0.044); Taz (*p* = 0.014) (Table 6). These calculations reveal individual differences in preferred contrafreeloading task types, similar to that observed in the Greys^13^.

#### Data summary

**Figure 1:**
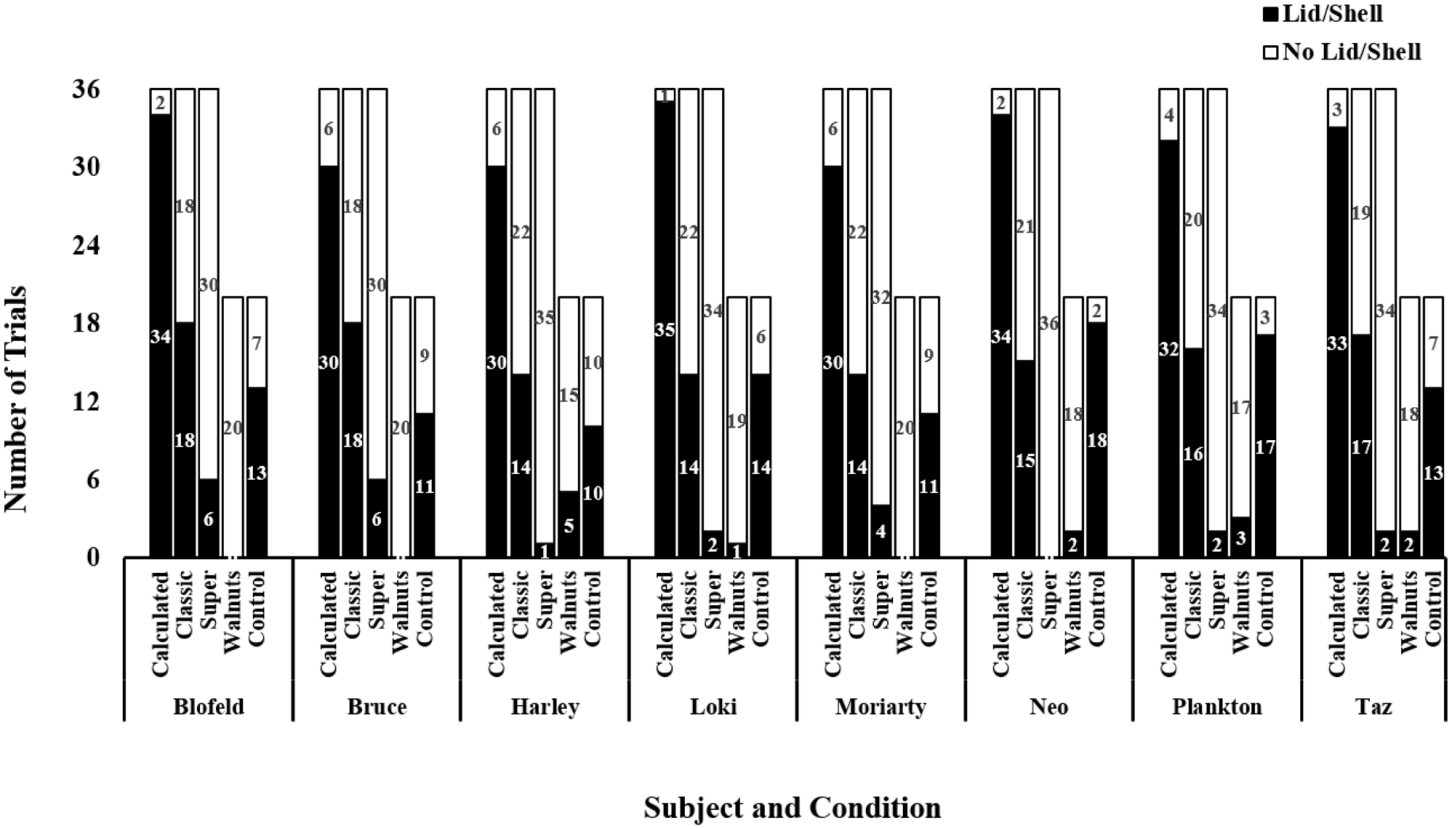
Contrafreeloading across kea and trial types.

The main results are as follows: The kea, like most of the Greys, performed only calculated contrafreeloading at a statistically significant extent. Although not to a statistically significant extent, qualitatively, the kea did engage in more classic contrafreeloading than the Greys: With the exception of one Grey, who classically contrafreeloaded at 87%, the remaining three Greys did so only 26–36% of the time; in contrast, all eight kea classically contrafreeloaded 39–50% of the time. The kea performed super contrafreeloading at levels comparable to the Greys. In Experiment 2, unlike the Greys, a subset of which engaged in nut contrafreeloading, kea showed little contrafreeloading. Figure 1 summarizes all contrafreeloading occurrences across all kea for both Experiments.

## Discussion

This study aimed to compare the extent of contrafreeloading in kea to that in Grey parrots, given that the two species exhibit very different levels of play: specifically, kea exhibit complex and frequent play^29,30,35,36^, whereas Greys exhibit considerably less play than several parrot species^29^. We found that, at the group level, although the overall amounts of kea classic contrafreeloading were nonsignificant, as a percentage of behaviour, kea generally contrafreeloaded more than Grey parrots in Experiment 1, whereas the opposite was true for Experiment 2. We compare the various behaviour patterns in detail, and propose explanations for our results below.

The most interesting comparisons for Smith et al.’s hypothesis are the results from classic contrafreeloading. In Experiment 1, kea performed this behaviour at non-negligible levels, given the supposed rarity of the behaviour^5^ (two birds at 50%; the others varying between 39 and 47%). In contrast, although one Grey did classically contrafreeload at a statistically significant level, the other three were at ≤ 36%. These data suggest that the kea may have found the task more engaging than did the Greys. However, given that only two kea chose to pop the lid of an empty cup in control trials significantly above chance, whereas three of the four Greys did so significantly above chance and one at chance, we doubt that the kea found the task inherently rewarding. We note that this comparison between both species must be interpreted cautiously due to differences in methodology: For the Greys, the control trials were performed at the end of the study, by which point they may have learnt to associate lid-popping with reward. However, the data from experimental trials in Smith et al.^13^ are such that their birds would have been primed in the opposite direction: For example, three of those four birds rarely chose the empty lidded cup when free food was available, nor did they classically or super contrafreeload to any significant extent^13^; an association-driven explanation is therefore unlikely. In contrast, the kea experienced this control condition at the start of the experiment, allowing them 20 trials to become acquainted with the affordances of both options that would be available throughout the study (lid-popping versus not lid-popping). This opportunity was important for kea, as this species has been previously shown to learn about object properties through extensive object manipulation^37^. That kea popped lids at or above chance in these first 20 control trials suggested two possibilities: (1) After these 20 trials, the task may have been familiar enough to no longer be of much interest (i.e., no longer novel and worthy of consideration) by the time rewarded trials began (recall nonsignificant downward trends for Harley Quinn and Blofeld). (2) They acquired some interest in popping the lids. This latter case seems more likely, as the lid-popping task still likely provided some added value. Kea engaged in non-negligible levels of classic contrafreeloading, such that the chance to pop a lid and eat could be considered more interesting than simply eating an identical but freely available reward. Furthermore, three kea chose a *lidded, empty cup* over a free, least-preferred reward at least half the time, again suggesting that the activity held some appeal of its own.

In Experiment 2 (which corresponds to classic contrafreeloading), all kea preferred freeloading for the walnut without a shell; two Greys, in contrast, nut contrafreeloaded at a statistically significant extent. This variability in behaviour at both the individual and species levels reveals the significance of a task’s proximate and potentially ultimate values in parrots’ choice to contrafreeload. Interestingly, although species like kea are hypothesized to prefer food items requiring high manipulation^38,39^, nut-cracking—chosen as an activity to provide direct comparison with the Greys^13^—is not prevalent in kea diet^40^, and that activity thus may not have been appropriate as an ethologically relevant one for kea. Greys, in contrast, are known to crack nuts in nature^41^. Future research could use a more ecologically relevant task for the kea, such as working to access food via digging or scraping^32^.

As with Smith et al.’s Greys^13^, kea in Experiment 1 performed calculated contrafreeloading to a statistically significant extent. All kea did so on over 83% of trials; for the Greys, three birds were close to 90% but one was at only 67%. Kea consistently selected their preferred food out of the two options provided, suggesting that the lid-popping action did not deter kea from selecting their preferred reward. In related trials, where the lid-status of food paired with an empty cup varied, kea, like some Greys^13^, preferred lidded food over an empty lidless cup, again showing that lid-popping for food was an acceptable task.

When examining situations in which food was discarded after contrafreeloading, we found that this choice in Experiment 1 was most common for Bruce. Notably, Bruce lacks a top mandible, making many of the manipulative behaviours more difficult to execute^42^. Bruce demonstrated consistent food preferences throughout the experiment, however, indicating that the reason some foods were discarded was, indeed, because they were too difficult for him to manipulate. In Experiment 2, Harley Quinn was the most likely to discard the nut, and did so exclusively in trials in which she chose the walnut without the shell (freeloaded). In these occasions, Harley Quinn was observed choosing the nut by tapping on it or the cup.

Like the Greys, the kea failed to super contrafreeload to a statistically significant extent. Furthermore, contrafreeloading trials in which a lid was popped but the food underneath was not consumed occurred most often with the least-preferred food. Given kea’s performance on control trials, the super contrafreeloading results are not surprising. Interestingly, when lid-status of food paired with an empty cup varied, some Greys very rarely—and depending on food desirability—preferred to pop the empty cup’s lid rather than consume the free food; as noted earlier, three of eight kea did so on at least half the trials when the food in the lidless cup was their least preferred option (sultanas). Both kea and Greys thus likely placed the appeal of the task along some “value scale” along with that of the available food rewards, the combination influencing their behaviour when the two variables were presented in various permutations. Notably, even in control trials, where no food was involved, no bird of either species found the task aversive, engaging in the behaviour at least 50% of the time. Future research could investigate how a different, more rewarding task would influence this balance and thus contrafreeloading for both species.

One possible alternative explanation for kea’s higher rates of contrafreeloading relative to those of Greys could be their natural tendency to probe and manipulate objects, thus causing them to pry off cup lids rather than manipulate lidless (open) cups. Were this action exploratory in nature, we would have observed significant decreases in behaviour as the experiment progressed, but note that we found no significant changes in any bird. Were they consistently drawn to lids and this behaviour were hard-wired, then we should have observed lid-popping appear significantly above chance across all three types of contrafreeloading. However, as discussed previously, kea did not *significantly* contrafreeload in the classic condition and actively *freeloaded* in super contrafreeloading conditions, suggesting that they were not simply interacting with lidded cups preferentially, but rather attending to the contents in the two cups and avoiding the additional manipulation of the lid when it led to a less (or, more often than not, equally) preferred food reward.

Another potential explanation for the differences observed between kea and Greys might be found in the theoretical overlap between contrafreeloading and play, and how individuals might view the contrafreeloading action as a type of play. As a seemingly nonfunctional, intrinsically motivating behaviour occurring in low-stress environments, incurring a positive mood, varying between conspecifics, and often incomplete and/or repeated^14,15^, play shares many proximate-level attributes with contrafreeloading^13^. Our results demonstrate that kea subjects inhabiting a low-stress, captive environment repeatedly chose to engage in classic contrafreeloading to a non-negligible extent and calculated contrafreeloading to a significant extent, varied in their behaviour between individuals, and at times, left the task incomplete (e.g., left food uneaten). Furthermore, evidence for intrinsic motivation to perform a given task is suggested by the kea’s overall differential behaviour between the two experiments, as well as inter-individual differences.

Importantly, this study serves only as a first step into determining whether play manifests as a form of contrafreeloading, but cannot ascertain that this is the only possible explanation for the presence or degree of contrafreeloading in the two species. Several alternative explanatory theories regarding the occurrence of contrafreeloading are enumerated in the discussion of Smith et al. (e.g., work ethic; information gathering; relief from boredom)^13^, and various other potential explanations (beyond playfulness) may reside at the species-level. Grey parrots (*Psittacidae*) and kea (*Strigopidae*) are separated by 50–80 million years of evolution^43^ and differ in their neurobiology (i.e., the size of the shell region related to vocal and possible cognitive abilities^44^). Differing ecological evolutionary pressures are also likely relevant: an island-based habitat^39^, a lack of natural predators^30,45^, and generalist diets^40,46,47^ are thought to have shaped the playfulness and cognitive abilities of kea^30,40,46,47^. Greys, in contrast, evolved predominantly on a continent (i.e., although they can be found on islands such as Principe, the Congo Grey is endemic to central Africa^48,49^), are subject to considerable predation^48,50–52^, and have a relatively less generalist diet (diverse but almost exclusively vegetarian and in which nuts play a significant role; see review in^50^). Such disparate evolutionary trajectories may offer other potential explanations for the differences in contrafreeloading observed between the two species, and future research could examine differences at genetic and/or neurological levels.

The varying rates of contrafreeloading observed between the species could have also been influenced by other factors. For example, although both parrot groups studied here inhabit enriched environments, are habituated to participating in experimental trials, and have access to food ad libitum, their habitats are markedly different. Notably, the Grey subjects live in “man-made” settings (i.e., Griffin and Athena in a lab; Pepper, Franco, and Lucci in private homes), whereas the kea inhabit a naturalistic zoo enclosure. Physical enrichment, although somewhat different in kind, is unlikely to have differed in quantity, as all birds are provided routine naturalistic foraging, and Lucci lives in a free-flight aviary. More likely is the difference in sociality: Relatively more subjects reside together in the kea group (15) compared to the Greys (two groups of two Greys and one Grey living with two birds of differing species), and thus variables such as social stimulation and flock-based foraging techniques could have contributed to the expression of contrafreeloading (note that subadult male kea are known to obtain food through kleptoparasitism^32^). In order to elucidate the role of habitat on contrafreeloading, future studies could examine the behaviour of species residing in more comparable captive conditions.

Future work should aim not only to apply these same methodologies to a broader range of parrot species, but also objectively quantify frequency and complexity of play across a wide range of parrots to allow a direct correlation between play and contrafreeloading over phylogeny in the parrot order. The apparent link between play behaviour and encephalisation in parrots^53^ offers another possible avenue for cross-species comparisons on contrafreeloading. Future research could also employ cognitive bias tests to quantify the mood of birds before and following contrafreeloading^54^, directly manipulate subjects’ participation in play behaviours or other control behaviours and observe whether engaging in play can increase contrafreeloading rates at the individual level, or perform behavioural coding of playfulness and/or arousal before and after contrafreeloading. Future research could incorporate more ecologically relevant contrafreeloading tasks to examine this behaviour at both the individual and species level, and approach the phenomenon by using both genetic and neuroscience techniques.

In sum, contrafreeloading is, by its very nature, an enigma whose study presents many difficulties. It varies across the diverse contexts within which it is studied, and given that it is rarely exhibited to a statistically significant extent, analyses that require comparing nonsignificant behaviour patterns across individuals and/or species is a challenging undertaking. Many explanations have been proposed, but contrafreeloading is still poorly understood, and its correlation with play is likely only one of several logical rationales. Nevertheless, our findings suggest that interest in play should not be discounted as a contributing factor.

## Methods

### Subjects and materials

**Figure 2:**
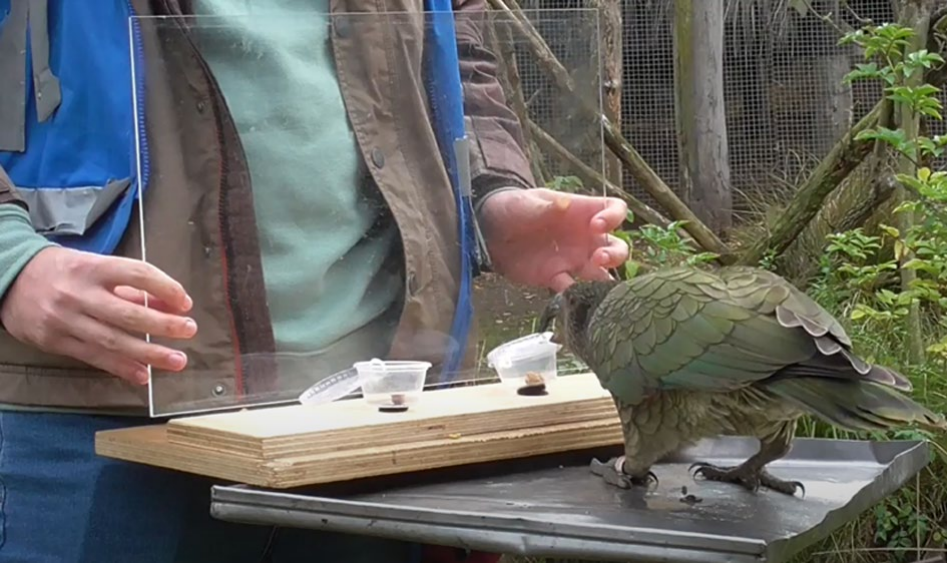
Kea subject performs calculated contrafreeloading during a test trial.

Subjects were eight adult kea aged 6 to 8 years (1 female) housed in a naturalistic outdoor enclosure at Willowbank Wildlife Reserve, New Zealand. Food and water were available ad libitum in the aviary, and subjects each received a daily test of five trials (Experiment 1) or three trials (Experiment 2), at least one hour after thei r morning feed. Subjects participated in the study voluntarily by coming to their individual platforms (42 cm × 42 cm) when called. Both experiments involved presentations of two food options, which could include 50 mm^3^ beef fat cubes, sultanas, or halved hazelnuts (Experiment 1), and shelled or unshelled halved walnuts (Experiment 2). These foods were not customarily provided as part of subjects’ daily diets but were all readily accepted and eaten at least ten times in a row by all individuals in tests prior to this study. As per preference tests also conducted prior to this study, fat cubes were determined to be the favourite food for all subjects, hazelnuts to be intermediate in value, and sultanas to be the lowest value food; subjects chose based on these preferences in 96.5% of food preference trials. All food items used within each experiment were of equivalent size and presented in 2-oz transparent plastic cups with removable transparent lids. The cups were attached to a small wooden board (45 cm × 13 cm × 1.5 cm) behind a Plexiglass screen (45 cm × 30 cm × 3 mm), which was removed so that subjects could make a choice and was then replaced after subjects selected one of the two cups (Fig. 2). The research was carried out with approval from the University of Auckland ethics committee (Reference Number 001816) and all methods were carried out in accordance with the relevant guidelines and regulations. The study was carried out in compliance with ARRIVE guidelines, which ensure ethical, transparent, and reproducible research with animal subjects^55^.

### Procedure

Procedures were replicated as closely as possible to those given Grey parrots in the original study^13^. Here, however, control trials to determine whether birds had preferences for either empty lidded or lidless transparent cups (i.e., for popping lids) were performed prior to experimental trials, rather than afterwards. This ordering ensured that kea’s natural preferences for this behaviour were tested from the start, rather than following experience with the experimental procedure. For all trials in the present study, a researcher blind to experimental hypotheses and wearing mirrored sunglasses stood across from the subject, who was separated from the cups by a Plexiglass screen. They showed each food item to the subject before placing it in a transparent plastic cup. The order in which the researcher’s hands covered the cups prior to presentation was counterbalanced and pseudorandomised across trials, given that subjects had had prior hand-tracking training^33,34^. After both options were briefly covered by the researcher’s hands, the Plexiglass screen was removed, allowing the subject to approach and make a choice by popping a lid and consuming the food. The screen was replaced immediately after consumption to avoid a second selection.

### Experiment 1

Prior to the experiment, subjects experienced 20 lid preference control trials to test for any individual preferences for an empty lidded versus an empty unlidded cup. Subjects were rewarded non-differentially between trials. Following these preference trials, subjects experienced combinations of food pairs at three levels of desirability (sultanas: low-value; hazelnuts: medium-value; and fat cubes: high-value) in lidded or unlidded cups. These pairs were divided into five categories over the course of 324 trials: (1) 144 food comparison trials, where both cups were either lidded or unlidded, but the food rewards differed, to determine whether food preferences remained stable throughout the experiment, (2) 36 calculated contrafreeloading trials, where one cup was lidded, the other unlidded, and the higher value reward was in the lidded cup, (3) 36 classic contrafreeloading trials, where again one cup was lidded, the other unlidded, but the rewards in both cups were identical, (4) 36 super contrafreeloading trials, where again only one cup was lidded, but now the higher value reward was in the unlidded cup, and (5) 72 empty comparison trials, where one cup was lidded and one of the two cups was empty. All possible food and lid combinations were presented 12 times, with both side placement of the closed lid and order of hand presentations counterbalanced across trials of the same type. All trials were presented interspersed in random order, which differed for every subject.

### Experiment 2

This second experiment investigated whether kea were more likely to exhibit classic contrafreeloading in a potentially more ecologically relevant food extraction task. To maintain the closest parallels with the Grey parrots, we used extracting a nut from its shell. That activity is not as ethologically relevant as one might expect in kea^40^, but would nevertheless provide additional comparisons at the species level. Here, subjects were given a halved walnut in the shell presented with the shell facing up, paired with a halved walnut without the shell. Walnuts were chosen as they constitute a part of their seasonal, although not daily, diet in captivity. Both rewards were placed in unlidded plastic cups in all trials; presentation followed the same protocol as Experiment 1. This experiment consisted of 20 trials and immediately followed Experiment 1.

### Analyses

All trials were coded in situ. Of the videotaped trials, 10% were randomly selected and coded by naïve observers; inter-observer reliability was 100%. Naïve observers also coded all the videos to determine whether subjects ate the food they selected; inter-observer reliability was 100%. For Experiment 1, subjects’ results in contrafreeloading trials—trial categories (2), (3), and (4) as described above—were analysed with logistic regression models. Between-model comparisons revealed that the best-fitting model for the data included test type (i.e. calculated, classic, or super contrafreeloading) only. Inclusion of trial number and subject ID into the model resulted in singular fits for the mixed-effects logistic regression models, so we opted for logistic regression models instead. Food comparison trials, trial category (1), were analysed in the same way, and in that case, the best fitting model included trial number, trial ID (the contents of both cups), and subject ID as a random effect with random intercepts. Finally, data from empty comparison trials from category (5) were best fitted by a model including test type (i.e., whether food was presented in the lidded or unlidded cup). Because preference controls and Experiment 2 comprised only 20 trials each, data were analysed at the individual level using two-tailed binomial tests (chance 0.5). Trials in which food rewards were not consumed were examined qualitatively, and comparisons between experiments were analysed using Fisher’s exact tests.

## Supplementary Information

Supplementary Information 1.

Supplementary Information 2.

Supplementary Information 3.

Supplementary Information 4.

## Data Availability

All data generated or analysed during this study are included in this published article [and its supplementary information files].
