# Author Correction: Contrafreeloading in kea (*Nestor notabilis*) in comparison to Grey parrots (*Psittacus erithacus*)

**DOI:** 10.1038/s41598-022-25501-x

**Published:** 2022-12-05

**Authors:** Gabriella E. Smith, Amalia P. M. Bastos, Martin Chodorow, Alex H. Taylor, Irene M. Pepperberg

**Affiliations:** 1 The Alex Foundation, 30 Curry Circle, Swampscott, USA; 2 Messerli Research Institute, University of Veterinary Medicine Vienna, Vienna, Austria; 3 School of Psychology, The University of Auckland, Auckland, New Zealand; 4 Department of Cognitive Science, University of California San Diego, San Diego, USA; 5 Department of Psychology, Hunter College, The City University of New York, New York, USA; 6 Department of Psychological and Brain Sciences, Boston University, Boston, USA; 7 Institut de Neurociències, Universitat Autònoma de Barcelona, Barcelona, Spain; 8 ICREA, Pg. Lluís Companys 23, Barcelona, Spain

Correction to: *Scientific Reports* 10.1038/s41598-022-21370-6, published online 18 October 2022

The original version of this Article contained errors in Table 1, where the first and third examples for the contrafreeloading type “Super” were incorrect. The correct and incorrect examples appear below.

Incorrect:

**Table:** 

Type	Definition	Example (choice of lidded/shell option)
Super	Performing an activity to access less-preferred food over preferred, free food	Fat (lidded) versus fat (unlidded) Sultana (lidded) versus hazelnut (unlidded) Sultana (lidded) versus sultana (unlidded)

Correct:

**Table:** 

Type	Definition	Example (choice of lidded/shell option)
Super	Performing an activity to access less-preferred food over preferred, free food	Hazelnut (lidded) versus fat (unlidded) Sultana (lidded) versus hazelnut (unlidded) Sultana (lidded) versus fat (unlidded)

The original Article has been corrected.

